# Maternal Death Review at a Tertiary Hospital in Ethiopia

**DOI:** 10.4314/ejhs.v31i1.5

**Published:** 2021-01

**Authors:** Matiyas Asrat Shiferaw, Delayehu Bekele, Feiruz Surur, Bethel Dereje, Lemi Belay Tolu

**Affiliations:** 1 Department of obstetrics and gynecology, Saint Paul's Hospital Millennium Medical College, Addis Ababa, Ethiopia

**Keywords:** maternal mortality, maternal death, Ethiopia

## Abstract

**Background:**

There is conflicting data on the rate and trends of maternal mortality in Ethiopia. There is no previous study done on the magnitude and trends of maternal death at Saint Paul's Hospital, an institution providing the largest labor and delivery services in Ethiopia. The objective of this study is to determine the magnitude, causes and contributing factors for maternal deaths in the institution.

**Methods:**

We conducted a retrospective review of maternal deaths from January 2016 to December 2017. Data were analyzed using SPSS version 20.

**Results:**

The maternal mortality ratio of the institution was 228.3 per 100,000 live births. Direct maternal death accounted for 90% (n=36) of the deceased. The leading causes of the direct maternal deaths were hypertensive disorders of pregnancy (n=13, 32.5%), postpartum hemorrhage (n=10, 25%), sepsis (n=4, 10%), pulmonary thromboembolism (n=3, 7.5%) and amniotic fluid embolism (n=3, 7.5%).

**Conclusion:**

The maternal mortality ratio was lower than the ratios reported from other institutions in Ethiopia. Hypertensive disorders of pregnancy and malaria were the leading cause of direct and indirect causes of maternal deaths respectively. Embolism has become one of the top causes of maternal death in a rate like the developed nations. This might show the double burden of embolism and other causes of maternal mortality that developing countries might be facing.

## Introduction

The World Health Organization's (WHO's) 10th revision of the international classification of diseases related health problems (ICD - 10) defines maternal mortality as “death of women while pregnant or within 42 days of termination of pregnancy irrespective of the site and size of pregnancy but related to or aggravated by the pregnancy or its management but not from accidental or incidental causes” (1).

The global estimate of maternal deaths in 2017 was 295,000(2). Sub-Saharan Africa and Southern Asia accounted for approximately 86% (254 000) of the estimated global maternal deaths in 2017 with sub-Saharan Africa alone accounting for roughly 66% (196 000) (2). Nigeria and India had the highest estimated numbers of maternal deaths, accounting for approximately one third (35%) of estimated global maternal deaths in 2017, with approximately 67 000 and 35 000 maternal deaths (23% and 12% of global maternal deaths), respectively. Three other countries also had 10000 maternal deaths or more: the Democratic Republic of Congo (16 000), Ethiopia (14 000) and the United Republic of Tanzania (11 000). Sixty-one countries were estimated to have had just 10 or fewer maternal deaths in 2017 (2).

Although the maternal mortality ratio is showing a declining trend, it remains still high. The 2016 EDHS (Ethiopian Demographic Health Survey) showed that the pregnancy related maternal mortality ratio of Ethiopia is 412 per 100,000 live births, while 2000, 2005 and 2011 EDHS reported maternal mortality ratio of 871/100,000, 673/100,000 and 676/100,000 live births respectively (3,4,5,6). A 2014 systemic review of both the community-based and hospital-based studies in Ethiopia has shown that there was no significant change in maternal mortality in about thirty years period (7).

The main causes of maternal mortality in Ethiopia are hemorrhage, anemia, r, infection, hypertension in pregnancy and obstructed labour (4, 5, 6). A systematic review of maternal mortality studies done from 1980 to 2012 in 18 health facilities in Ethiopia has shown the following findings. In the first 20 years of the study, abortion (31%), obstructed labor (29%), sepsis (21%) and hemorrhage (12%) were the major causes of maternal deaths. The most significant causes of maternal death in the final 10 years of the study period were, however, obstructed labor (36%), hemorrhage (22%), pregnancy-induced hypertension (19%) and sepsis (13%) (8).

An analysis of causes of maternal mortality by residence among 39 maternal deaths at two hospitals indicated that most of the abortion (2 of 3) and ruptured uterus (3 of 4) deaths were coming mainly from outside of Addis Ababa. However, most of the maternal deaths due to eclampsia (12 of 13), hemorrhage (5 of 7), infection (all 3) and pulmonary embolism (all 3) were from Addis Ababa (9).

There is the paucity of published works on maternal mortality in Ethiopia where there is considerable maternal mortality. No published study has been conducted in Saint Paul's Hospital Millennium Medical College (SPHMMC) since it was established, although it is currently one of the centers with the highest number of deliveries in the country. In-depth local studies can help in suggesting strategies and in decision making to decrease the rate of maternal mortality in the institution as well as in the country.

The objective of this study was, therefore, to systematically analyze the maternal deaths at SPHMMC to determine the magnitude, causes and contributing factors for maternal mortality at the institution.

## Materials and Methods

A prospective cross-sectional study was conducted from January 2016 to December 2017 at Saint Paul's Hospital Millennium Medical College in Addis Ababa, the capital city of Ethiopia. All maternal deaths during the study period were included in the study.

We used the WHO maternal death definition to include maternal deaths. The death was considered as direct maternal death if it resulted from obstetrical complications of the pregnancy state, labor, or puerperium. Indirect maternal death was an obstetrical death resulting from a disease previously existing or developing during the pregnancy, labor, or puerperium; death was not directly due to obstetrical causes but might be aggravated by the physiologic effects of pregnancy. A non-maternal death, defined as an obstetrical death resulting from accidental or incidental causes unrelated to the pregnancy or its management, was excluded from the study.

The data collection instrument was adapted from the Maternal Death Surveillance and Response Technical Guidelines of Ethiopia (10). Using registration numbers of maternal deaths, the case files were retrieved from the medical record department of the hospital within 24 hours of the occurrence of maternal deaths. To ensure the quality of data, three data collectors were trained and pre-tested the checklist tool. All data were collected and stored anonymously. Supervisors cross-checked for completeness and accuracy of the data every week. Data were analyzed using SPSS version 20.

A formal letter of approval was obtained from Saint Paul's Hospital Millennium Medical College Ethical Review Committee. Permission to conduct the study was obtained from the hospital administration. Confidentiality was maintained during data collection, analysis and interpretation.

The independent variables were age, place of residence, level of education, occupation, marital status, parity, antenatal care status, place of delivery, gestational age of the pregnancy and mode of delivery. Maternal death was the dependent variable.

## Results

Data were collected from 1 January 2016 to 31 December 2017. In total, 40 maternal deaths that occurred during the study period were included in the study. During the same period, 17,522 live births made the maternal mortality ratio (MMR) of the institution 228.3 per 100,000 live births.

The mean (± SD) maternal age at the time of death was 27.85 (±5. 56) years, and the majority (72.5%) of the cases were between the age group of 20–34 years. More than two-thirds of the mothers (70%) came from outside of Addis Ababa while the women had an average of 3.28 (±2. 86) pregnancies, the average parity was 2.73 (±2. 77) (Table 1). Thirty-four (85%) of the cases had a minimum of one antenatal care (ANC) visit while the remaining 6(15%) had no antenatal care visit in the index pregnancy. Twelve (30%) of those with ANC had their last visit to the hospital (Table 1).

**Table 1:** Characteristics of maternal deaths at SPHMMC from January 2016 to December 2017

Variables		No (%)
**Age group**	<20	1 (2.5)
	20–34	29 (72.5)
	>34	10 (25)
**Place of residency**	Addis Ababa	12(30)
	Outside Addis Ababa	28 (70)
**Gravidity**	Primigravida	17 (42.5)
	2–4	10 (25)
	≥5	13 (32.5)
**Parity**	0	6 (15)
	1–4	24 (60)
	≥5	10 (25)
**ANC booking status**	ANC at Health center	22 (30)
	ANC at Hospital	12 (55)
	Did not have ANC	6 (15)

Most of the pregnancies (90%) had reached the third trimester of the pregnancy while three deaths (7.5%) happened in the second trimester of pregnancy. There was also a single maternal death in the first trimester of pregnancy (Table 2). Three-quarter of the deaths (31, 77.5%) occurred in the postpartum period. Eighteen (45%) mothers died in the first 24 hours of the postpartum period (Table 2). There were two home deliveries and the rest of them were delivered at a health facility. Eighteen (45%) of the mothers delivered by cesarean section, and two delivered by obstetrics forceps. There were 22 live births and 11 stillbirths from the deliveries. Three perimortem cesarean sections were performed during the study period, of which one resulted in a live birth.

**Table 2:** Circumstances of the maternal deaths at SPHMMC from January 2016 to December 2017

	Variable	No (%)
**Gestational age**	1^st^ trimester	1 (2.5)
	2^nd^ trimester	3 (7.5)
	3^rd^ trimester	36 (90)
**State of pregnancy at death**	Antepartum	6 (15)
	Intrapartum	1 (2.5)
	Postpartum	31 (77.5)
	Post-abortion	1 (2.5)
	Post molar pregnancy	1 (2.5)
**Timing of death after arrival to**	Death within 60 minutes arrival	11 (27.5)
**the hospital**	Death after 60 minutes arrival	29 (72.5)
**Reason for referral**	For better diagnosis and management antepartum	14 (35)
	Eclampsia	6 (15)
	Postpartum hemorrhage	6 (15)
	Molar pregnancy	1 (2.5)
	Uterine perforation after MVA* for abortion	1 (2.5)

* manual vacuum aspiration

Twenty-eight (70%) of the mothers were referred. The reasons for referrals included eclampsia and postpartum hemorrhage, each accounting for 21.4% of the referrals. Fourteen mothers (35%) were referred to antepartum from the catchment health centers of the hospital for better evaluation and management. More than a quarter of the maternal deaths occurred within one hour of arrival to the SPHMMC with a referral from other health facilities (Table 2).

Thirty-six (90%) of the deceased suffered direct maternal deaths, and four (10%) died from indirect causes. The leading causes of the direct maternal deaths were hypertensive disorders of pregnancy (n=13, 32.5%), postpartum hemorrhage (n=10, 25%), sepsis (n=4, 10%), pulmonary thromboembolism (n=3, 7.5%) and amniotic fluid embolism (n=3, 7.5%). Pulmonary thromboembolism was diagnosed by chest CT scan while diagnoses of amniotic fluid embolism was made clinically. One woman died from complications of safe abortion. She was referred to the hospital for uterine perforation after manual vacuum aspiration done for safe pregnancy termination. There was also a single death from hyperemesis gravidarum complicated with hypokalemia resulting in cardiac arrest at 17^th^ weeks of gestation. One mother died following anesthesia complications, high spinal anesthesia, during the cesarean section. Ten (25%) of the hypertensive disorders of pregnancies had eclampsia. From the postpartum hemorrhage, 8(20%) were due to uterine atony and two of them (5%) had genital trauma. There was no single maternal death due to antepartum hemorrhage and obstructed labor during the study period (Table 3).

**Table 3:** Causes of maternal death at SPHMMC from January 2016 to December 2017

Causes of maternal death	No (%)
**Direct maternal death**	36 (90)
**Hypertensive disorder of pregnancy**	13 (32.5)
**Postpartum hemorrhage**	10 (25)
**Sepsis**	4 (10)
**Amniotic fluid embolism**	3 (7.5)
**Pulmonary thromboembolism**	3 (7.5)
**Abortion**	1 (2.5)
**Hyperemesis gravidarum with hypokalemia**	1 (2.5)
**High spinal**	1 (2.5)
**Indirect maternal death**	4 (10)
**Malaria**	2 (5)
**AIDS-related**	1 (2.5)
**Thyroid storm**	1 (2.5)

From the indirect cause of maternal deaths, two (5%) were from malaria. A single maternal death resulted from thyroid storm that developed following total abdominal hysterectomy done for molar pregnancy with thyrotoxicosis. AIDS-related cause accounted for one of the indirect maternal deaths (Table 3). When we see the causes of the maternal deaths using the three-delay model (11), six (15%) of them had a delay in seeking care, seven (17.5%) had a delay in reaching the right health facility and twenty-seven (67.5%) had delay within the health facility.

## Discussion

The maternal mortality ratio of Saint Paul's Hospital was 228.3 per 100,000 live births during the study period. This was lower than the 287.5/100,000 live births MMR seen in a university teaching hospital report in Cameroon (12). It was also lower than the Ethiopian institutional MMR reported from Ayder Comprehensive Specialized Hospital and Jimma University Specialized Hospital which were 569.7 and 350 per100,000 live births respectively (13,14). It is also much lower than the 353 per 100,000 live births institutional MMR of Ethiopia (15). This observed relatively lower maternal mortality could be due to better health service and improved quality of obstetric and gynecologic care because of the expansion and commencement of different specialty and subspecialty training in the hospital.

Hypertensive disorders of pregnancy, most of whom had eclampsia, took over postpartum hemorrhage as the leading cause of direct maternal deaths. This is like the findings in a Nigerian hospitals (16,17). This differs from most local, sub-Saharan Africa, and global findings where hemorrhage is the most common cause (13,14,18). There was no death due to antepartum hemorrhage in our study. This might show that the hospital has fared better concerning managing hemorrhagic complications. However, the further reduction in maternal death can still be achieved by improving blood transfusion services, as observed in high-income countries (19).

There was no death due to obstructed labor during the study period. There was also no maternal death from obstructed labor from a teaching hospital in Mekelle, Northern Ethiopia (13). This is in sharp contrast to a study in Jimma, Southwestern Ethiopia, where uterine rupture caused 8.3% of the maternal deaths in the institution (20). This might indicate an overall decrease in the incidence of obstructed labor and better obstetrics service provision in the country.

Embolism (clot and amniotic fluid) has become one of the top causes of maternal death accounting for 15% of maternal deaths. This is like the 13.8% reported from developed nations (18). Yet, this is much lower than the rate reported from local studies (13,14). This might show that embolism becomes a more prominent factor as other causes of maternal mortality decreases.

Abortion is one of the least common causes of maternal death in our study. This is consistent with the significant decrease in abortion-related maternal death in Ethiopia (8). This is explained by improved access to safe abortion services following the liberalization of abortion in the country.

Indirect cause accounted for one in ten maternal deaths. This was like 10% reported from Northern Ethiopia in 2013 (21) but was lower than sub-Saharan and global studies which were 28.6 and 27.5% respectively (18).

Only one of the forty maternal deaths were aggravated by AIDS in our study. This contrasts with the overall proportion of HIV related maternal deaths in sub-Saharan Africa, 6.4%, which is highest in the world (18). However, the finding was consistent with other studies done in Ethiopia (13,22). Half of the indirect maternal deaths were caused by malaria. This shows the critical importance of the prevention of malaria in pregnant women in endemic areas.

As shown previously in other studies (22,23), the postpartum period is the time when most of the maternal deaths occur. Therefore, we emphasize the importance of quality obstetric services in health facilities during this period for women attending facility delivery.

When we see the maternal deaths in the three-delay model (11), two-third (67.5%) had delays within the health facility. This might show that improvement in the care provided in the health facilities, both at health centers and hospital levels, is an important target to decrease maternal mortality.

More than a quarter of the maternal deaths occurred within one hour of arrival to SPHMMC with a referral from other health facilities. This might show that critical time for care may be wasted during the transition from the health facilities to the hospital. This gap can be targeted by improving the referral system, which includes accompanying the mothers by qualified health professionals in emergency care and better-equipped ambulance for critical care.

Given the high number of mothers arriving in a critical condition in the study, this excess burden suggests significant deficiencies in the prevention, identification, and referral of severe morbidities at lower care levels and private health facilities, as well as the presence of community-level barriers to healthcare-seeking (24).

The study's largest strength lies in its being a prospective study. We collected the data within 24 hours of the occurrence of maternal death. This helped us to have a wealth of data about each maternal death circumstance. Despite our efforts to ensure accurate implementation of the study protocol and high-quality data, medical protocols and records formats may have resulted in misclassification and affected the timing of some obstetric events and interventions. We also did not have autopsy after the maternal deaths to confirm the possible cause of death. The possible cause of death was determined based on the clinical impression of the obstetrician and gynecologist who evaluated and provided clinical care for the woman before her death.

In conclusion, we found relatively lower maternal mortality at Saint Paul's Hospital in comparison to similar institutions in the country. Hypertensive disorders of pregnancy and malaria were the leading cause of direct and indirect causes of maternal deaths respectively. Embolism has become one of the top causes of maternal death in a rate like the developed nations. This might show the double burden of embolism and other causes of maternal mortality that developing countries might be facing. More than a quarter of the maternal deaths occurred within one hour of arrival to the hospital and two-third of the deaths had a tertiary delay at different health facility levels. The postpartum period is the time when most of the maternal deaths occurred. The finding suggests the need for strict antepartum screening for preeclampsia and malaria for early identification and management at all levels of care. Stakeholders should consider reorientation of referral management of cases and improving the high dependency and the intensive care units for critically ill mothers. The fact that embolism becomes one of the top causes of maternal deaths suggests the need to develop national protocols that guide the application of antepartum and postpartum thromboprophylaxis. Finally, we recommend a multicenter study on maternal deaths to determine patterns of causes for context-specific intervention.
